# A 60-year-old female with giant retroperitoneal liposarcoma

**DOI:** 10.11604/pamj.2021.39.77.29777

**Published:** 2021-05-26

**Authors:** Danilo Coco, Silvana Leanza

**Affiliations:** 1 Department of General Surgery, Ospedali Riuniti Marche Nord, Pesaro, Italy,; 2 Department of General Surgery, Carlo Urbani Hospital, Jesi, Ancona, Italy

**Keywords:** Giant, retroperitoneal, liposarcoma

## Image in medicine

Retroperitoneal tumors give non-specific symptoms until they have reached a substantial size. Liposarcoma and leiomyosarcoma are the most common retroperitoneal carcinoma amount of 70% and 15% respectively. Hodgkin´s and non-Hodgkin lymphoma and epithelial tumors or metastatic disease are less common. We present a 60-year-old female who was referred to the Emergency Department with acute onset of abdominal distension, nausea, vomiting, right back pain and lipothymia. She had a medical history of essential hypertension and diabetes mellitus. On physical examination, she had a good performance status, arterial blood pressure was 90/60 mmHg, pulse rate was 120 b/min, oxygen saturation of 93%. Blood exams demonstrated no lymphocytes 4x 103/mmc, hemoglobin 7g/dl. Physical examination of the abdomen revealed a giant non-tender mass in the left and midline quadrants. The patient was transfused with 4 units of blood. Abdomen computed tomography (CT) scan revealed a retroperitoneal mass of 35 cm involving the stomach, the spleen, the kidney and surrounding the vena cava (A). He was referred to a Multidisciplinary Team (MDT) to perform a CT scan Fine-Needle-Aspiration (FNA) which confirms a well differentiated liposarcoma. A laparotomy was performed with total excision of retroperitoneal mass (B). At 6^th^ day, the patient was in good clinical condition and he was discharged. Hystopathological finding: well differentiated liposarcoma. French Federation of Cancer Centers Sarcoma Group: Grade 1 tumor differentiation: 1; mitotic count: 1; tumor necrosis: 0 (C).

**Figure 1:**
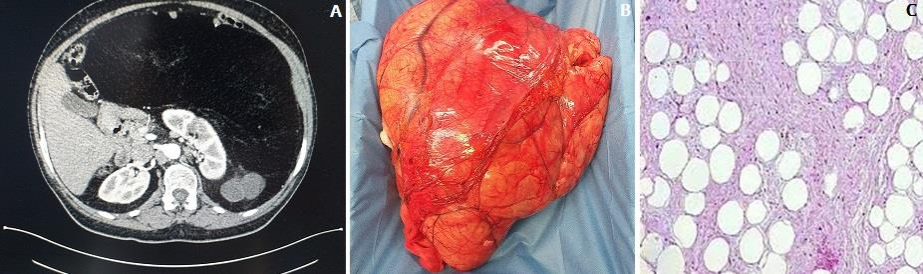
abdomen CT scan revealed a retroperitneal mass of 30 cm involving the stomach, the spleen, the kidney and surrounding the vena cava (A); total excision of retroperitoneal mass (B); well differentiated liposarcoma, French Federation of Cancer Centers Sarcoma Group: Grade 1 Tumor differentiation: 1; Mitotic count: 1; Tumor necrosis: 0 (C)

